# A Service-Oriented Healthcare Message Alerting Architecture in an Asia Medical Center: A Case Study

**DOI:** 10.3390/ijerph6061870

**Published:** 2009-06-17

**Authors:** Po-Hsun Cheng, Feipei Lai, Jin-Shin Lai

**Affiliations:** 1 Department of Software Engineering, National Kaohsiung Normal University, No. 62, Shenjhong Road, Yanchao Township, Kaohsiung County, 82444, Taiwan; E-Mail: cph@nknu.edu.tw; 2 Superintendent Office, National Taiwan University Hospital, No. 7, Chung-Shan South Road, Taipei, 10002, Taiwan; E-Mail: flai@ntu.edu.tw; 3 Department of Physical Medicine and Rehabilitation, National Taiwan University Hospital and College of Medicine, National Taiwan University, No. 7, Chung-Shan South Road, Taipei, 10051, Taiwan

**Keywords:** alert, disease surveillance, health information system, information technology, patient safety, service-oriented architecture, short message service

## Abstract

This paper illustrates how our development team has used some information technologies to let physicians obtain an instant abnormal laboratory result report for critical patient care services. We have implemented a healthcare message alerting system (HMAS) on a healthcare short message service (HSMS) engine and the distributed healthcare-oriented service environment (DiHOSE) in the National Taiwan University Hospital (NTUH). The HSMS engine has a general interface for all applications which could easily send any kind of alerting messages. Fundamentally, the DiHOSE uses HL7 standard formats to process the information exchange behaviors and can be flexibly extended for reasonable user requirements. The disease surveillance subsystem is an integral part of NTUH new hospital information system which is based on DiHOSE and the disease surveillance subsystem would send alerting messages through the HSMS engine. The latest cell phone message alerting subsystem, a case study, in NTUH proved that the DiHOSE could integrate the user required functions without much work. We concluded that both HSMS and DiHOSE can generalize and extend application demands efficiently.

## 1. Introduction

In 1999, Collen [1] introduced a vision of healthcare and informatics in 2008 powered by evolving information technologies that both enabled and benefited from widespread mergers and acquisitions of healthcare enterprises. With widespread numbers of patients receiving services in numerous, geographically scattered facilities, these amalgamated enterprises required a sophisticated information infrastructure to balance support for patient care, cost containment, and quality control. In order to illustrate the related researches and implementations completely, we describe herein three subsections of this technology: the laboratory alerting systems, advanced medical services, and health information standard adoption.

### 1.1. Laboratory Alerting Systems

The patient status monitoring and disease surveillance would be achieved through a changeable healthcare alerting system. In 1989 Bradshaw *et al.* [2] proposed a Computerized Laboratory Alerting System (CLAS) based on the HELP medical information system at the LDS hospital to track and alert of the existence of life-threatening conditions in hospitalized patients which are indicated by laboratory test results.

In CLAS, the initial average alert response times at nursing divisions ranged from 5.1 to 58.2 hours. The average alert response time dropped to 3.6 hours when a review of alert conduct was integrated with laboratory result reviews, and to 0.1 hour after installation of a flashing light to notify hospital staff of the presence of new alerts. Furthermore, Modai, *et al.* [3] enhanced the patient management services for increasing the response time of clinical staff and saving more lives in 1999.

Some other special computerized alerting cases have constantly proved the efficiency of these systems. For example, Hoch, *et al.* [4] and Atreja, *et al.* [5] focused on potassium test alerting in 2003. In the meantime, Oniki, *et al.* [6] examined the effect of computer-generated reminders on the nurse charting deficiencies in two intensive care units. Then, in 2005 Kucher, *et al.* [7] used automatic alerts to prevent venous thromboembolism among hospitalized patients.

### 1.2. Advanced Medical Services

However, information technology is changing quickly. For example, Kuperman *et al.* [8] enhanced two advanced alerting features in 1997. The first feature identifies and displays the relevant data field between the login times of a specimen led to an alerting result and the time the alert is reviewed. The second feature retracts alerts when the alerting values are edited and no longer satisfies the alerting criteria.

Another applied information technology for alerting mechanisms, web messaging and e-mail [9], can be utilized to improve the relationship between physicians and patients. For example, in 2003, Liederman, *et al.* [10] and Dunbar, *et al.* [11] coincidently depicted new ways to improve physician and patient communications with each other via web messaging. On the other hand, Lenert, *et al.* [12] used an e-mail messaging system to ameliorate quit rates in an Internet smoking cessation intervention in 2004.

Furthermore, Fritsche, *et al.* [13] used a case-based reasoning algorithm with dynamic time warping as the measure of similarity that allows delay of the use of mandatory laboratory alerting systems to conditions in which abnormal laboratory results of the norm and critical states can be detected only by recognition of pathological changes over time. Fundamentally, most of the advanced alerting features can be integrated with clinical information systems into an extensive clinical decision support system [14,15]. In 1999, Randolph *et al.* [16] categorized the functions of computer-based clinical decision support systems into eight functions: alerting, reminding, critiquing, interpreting, predicting, diagnosing, assisting, and suggesting.

This is a possible way for the profession to expand information technology into the public health field. For example, a team of informatics and public health specialists from Utah and Pittsburgh implemented the Real-time Outbreak and Disease surveillance (RODS) system in Utah for the 2002 Olympics Winter Games [17]. Moreover, the National Retail Data Monitor (NRDM) [18] in the USA receives data from 10,000 stores which sell healthcare products, including pharmacies. The high volume of automatic retail sales data enables the Monitor to congregate information from thousands of store locations in near to real time for use in public health surveillance.

### 1.3. Health Information Standard Adoption

Note that both RODS and the NRDM are automatic monitoring systems that unintentionally created information technologies efficiency. Furthermore, the RODS also utilized a Health Level Seven (HL7) standardized message protocol to communicate among hospitals and clinical encounters [19]. Conceptually, Randolph’s categories are a kind of standard for understanding the unclear defined medical terminologies and they help information engineering to develop applicable clinical information systems [16]. Similar reports emphasizing the need for standardization were made in 1899 [20] and 2005 [21]. Consequently, applicable standardized health care flows, data, and procedures will reduce misunderstandings, improve the client safety, and increase the quality of healthcare.

## 2. Problem Statement

In our hospital, we roughly divided all of our production systems into several distinct generations. The first generation systems are the IBM mainframe systems, which were decommissioned in 2007. The second generation systems were adopted the client/server technologies. The third generation systems utilize traditional 3-tier web-based methodology and the fourth generation systems derive service-oriented architecture (SOA) with N-tier Web Services under Layer 4 load balanced solution.

At NTUH the laboratory information systems (LIS) and hospital information systems (HIS) fall under the third and the fourth generation, respectively, although the processing roles for LIS and HIS are quite different. The LIS is responsible for the specimen tests in the laboratories. On the other hand, the HIS takes care of the outpatient, inpatient, and emergency patient processes in the hospital. Both HIS and LIS can be integrated with each other by either loosely-coupled or tightly-coupled style.

Originally, there were many reporting information systems to support daily clinical services at NTUH. They included the LIS, the radiology information system, observation information system, etc. Most of them were implemented one by one since 1995 with distinctive information technologies. Manifestly, the longer the execution period of the information systems, the broader and deeper applied functions resulting from the user requirements are accumulated. Meanwhile, the complexity of programming maintenance of the information systems progressively increased correspondingly.

The LIS is a high productivity legacy information reporting system which was implemented at NTUH from 1985 to 2002 and has gone through three different generations of information technology development. The latest and third generation LIS was implemented in 2002 and is still in service [22,26]. It serves at least 30 departments and connects about 50 medical instruments. The laboratory technicians and doctors submit thousands of results of diverse laboratory items every day. The amount of these laboratory items is so voluminous that quality assurance is one of the key tasks for laboratory worker.

Generally speaking, the laboratory workers continue to meet acceptable laboratory quality control limits and this allows most of the clinical doctors trust the laboratory results. Additionally, the clinical doctors will request further services from the laboratory department after they fully trust the laboratory results. Some of the physicians frequently want to obtain some remarkable laboratory results as soon as possible and start a new treatment for specific critical patients. Manifestly, it is a problem for our information engineers to try to utilize some information technologies to meet such physician requirements for critical patient care services in our medical center. Furthermore, our development team also tried to further integrate our fourth generation HIS, which was implemented from 2003 to 2007 [23,28] and the third generation LIS.

## 3. State of the Art

**Figure 1.**
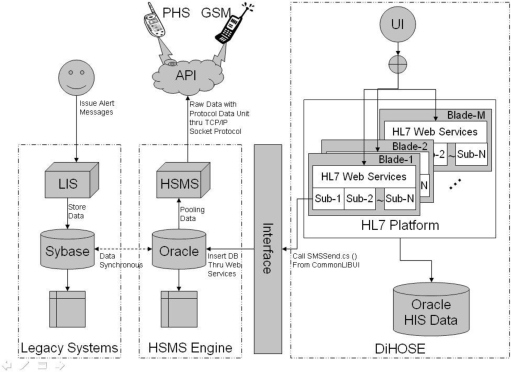
Integrated data flows for heterogeneous environment.

In order to recommend a flawless resolution to satisfy the clinical requirements and based on the CLAS-like [2,3] ideas, National Taiwan University Hospital (NTUH) plans to implement the healthcare message alerting system (HMAS) with the healthcare short message service (HSMS) engine based on the distributed healthcare-oriented service environment (DiHOSE) and integrated with the legacy reporting systems in 2005. The following paragraphs illustrate the legacy reporting systems, the DiHOSE, and the HSMS engine, the HMAS, respectively, as presented in Figure 1.

## 4. Proposed Solution

We propose our DiHOSE methodology to face the challenge of meeting physicians’ critical requests and integration of the second generation LIS, the third generation LIS, and the fourth generation HIS, because some connections in our medical instruments were not fully upgraded from the second generation to its third generation. This is why those alterations are needed within our proposed solution. The following paragraphs illustrate our proposed solution to solve the problem which is stated previously. There are three distinct sections which relate to the state of the art section and describe as below.

### 4.1. DiHOSE

Based on Collen’s vision [1], NTUH created the DiHOSE, which was a specific healthcare information system environment to assist multifunctional medical services with a distributing operating mechanism. NTUH activated the DiHOSE to implement its outpatient information systems in 2004. In the meantime, the DiHOSE can support other information system implementations, such as in-patient information systems, emergency information system, etc.

The DiHOSE has multi-blade servers with layer four (L4) switches to appropriately incorporate the message requests from thousands of client sides. That is, it can be dynamically scaled by adjoining appropriate numbers of blade servers to elevate the execution capability. Moreover, it is constructed with N-tier architecture and can depend on the server loadings to dynamically adjust the number of blade servers between application servers and web servers.

One of the international healthcare information exchange standards, HL7, was adopted and acts as a standard message core inside the HL7 platform of the DiHOSE, which is cooperatively the line of the RODS implementation [17,19]. The HL7 platform includes multi-blade servers and each server is installed with the same healthcare subsystems. That is, there are 14 subsystems inside each blade server. The subsystems contain registration, diagnosis, clinical process, pharmacy, laboratory, observation, radiology, nuclear magnetic, therapy, scheduling, disease surveillance, billing, insurance, credit, etc.

### 4.2. HSMS

The short message service (SMS) is one of the latest popular communication services for cell phones. NTUH properly activates the SMS to implement the HSMS engine and dedicate it to healthcare enterprise usage. The short message (SM) is transferred from the legacy reporting systems, LIS, or web applications to the short message service center (SMSC) of the telecommunication companies via Internet. Short messages sending statistical reports can also be read from the HSMS engine.

After studying some research articles, we can roughly categorize at least two kinds of the application developing methodologies: legacy application development methodology (LADM) [24–26] and web application development methodology (WADM) [27–29]. The following paragraphs illustrate LADM, WADM, HSMS and their composite integration process.

The LADM is a database replica method and indirectly integrates the 2-tier client/server information systems. For example, the LIS of NTUH manipulates a Sybase relational database to accumulate the laboratory results. The laboratory workers screen all of the results every day. If workers find any extraordinary laboratory result, they will specify and transmit a specific SM to a specific clinical doctor. However, the above process only momentarily deposits the SM in the SM table of the LIS database for the HSMS engine to do additional processing. That is, the HSMS engine is continuously polling the SM table of the LIS and transmitting the SM out. Besides it routinely refreshes the status code in the SM table of LIS.

The other alternative development methodology, WADM, is an N-tier development methodology and uses Web Services to automatically treat the data messages. Fundamentally, WADM implements a web user interface to partially manipulate the client processes and transmit the SM with CommonLibUI application programming interface (API) via the HSMS engine instantaneously. The HSMS engine and web user interface are enforced by kindred information technologies and are seamlessly integrated with each other. Therefore, it is evident that the turn-around time of the WADM is shorter than one of the LADM.

Moreover, the HSMS engine can transmit SM to the different cell phone systems of different telecommunication companies in Taiwan. For example, if the receiver utilizes the Personal Handy-phone System (PHS), the HSMS engine calls the PHS system API which is supported by the First International Telecom (FITEL). From another point of view, if the receiver uses a Global System for Mobile communication (GSM) cell phone, the HSMS engine calls the GSM system API which is supported by the Chunghwa Telecom Co. Ltd. (CHT). Therefore, the HSMS engine has a comprehensive interface for all subsequent implementations which could certainly send any kind of alerting messages.

### 4.3. HMAS

**Figure 2.**
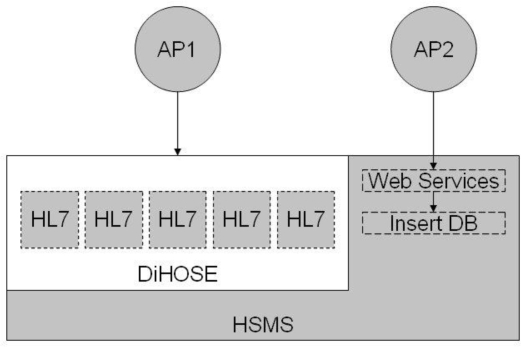
Relation between DiHOSE and HSMS.

Based on the conceptions of [4–7], the HMAS is implemented on an HSMS engine and by virtue of the DiHOSE in NTUH, as illustrated in Figure 2. NTUH utilizes both LADM and WADM to integrate the legacy reporting systems, LIS, and the web applications. Fundamentally, the DiHOSE uses HL7 standard formats to process the crucial data exchange behaviors. The DiHOSE can flexibly incorporate practical user requirements, since it uses the international healthcare standard message format. The disease surveillance subsystem is an integral part of the fourth generation HIS at NTUH. It is based on DiHOSE and the disease surveillance referred HL7 messages would send an alerting message through the HSMS engine and simply cooperate with the other subsystems. That is, we utilize a 4-tier architecture which mixes with 3-tier web-based solution to integrate all of the heterogeneous systems with HL7 subsystem capsulation [23].

The capsulation choice will be based on the user requirements. For example, the smart card communication interface with desktop computer will connect a smart card reader with a USB 2.0 line and such a connection processing will be simply implemented with a 4-tier ActiveX component window to process related tasks. The smart cards include at least the Taiwan National Health Insurance (NHI) card and any kind of credit card.

## 5. Results and Discussion

In order to implement the HMAS, NTUH integrates the legacy reporting systems and HSMS engine into the DiHOSE. That is, NTUH appends some functions of the surveillance subsystem in DiHOSE to interface with the HSMS indirectly. What appear in the paragraphs below specifies the described processing procedures and the user interface of the legacy reporting systems, LIS, and the HMAS, individually.

**Figure 3.**
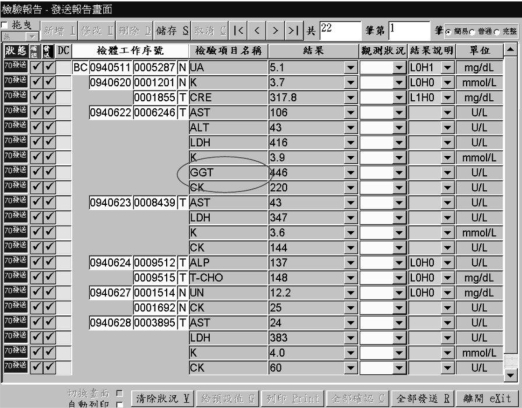
Specify laboratory item to alert doctor in legacy reporting system.

For example, the laboratory technician browses through the laboratory reports from LIS and expressly notes that the laboratory item, GGT, exceeds the upper limit of the predefined alerting boundary in Figure 3. This figure represents a traditional Chinese laboratory report release form which lets a technician review the laboratory report before data submission.

Most of the data window manipulation operations are located at the top command line which at least includes drag, insert, update, delete, save, cancel, first row, previous row, next row, last row, total rows, which row, easy mode, common mode, and complete mode, respectively, from left to right. Meanwhile, the data window lists data with titles from left to right, such as process status, confirmed flag, released flag, delta check flag, specimen sequence, specimen item name, value, observation status, value description, and unit, respectively. At the bottom of this window is another data manipulation command line which includes from leftmost to rightmost screen switch, automatic printing, clear status, set up default value, printing, confirm all, release all, exit this window, respectively.

**Figure 4.**
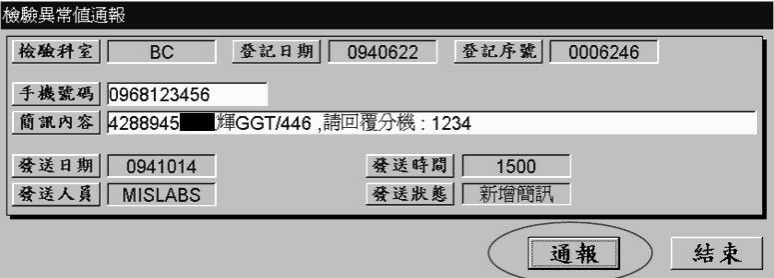
Confirm short alerting message in legacy reporting system.

In order to alert the clinical doctor as early as possible, the specialist double clicks the GGT item and successfully launches the alerting windows which contains the appropriate information. Then he transmits the concise alerting message after he confirms the clinical doctor’s cell phone number and message content in Figure 4.

Figure 4 is also a traditional Chinese laboratory abnormal report alert form which lets a technician confirm and process the specific abnormal report outcome. The data fields at the first line are department abbreviation, login date, and login number. The data fields at the second and third line are cell phone number and short message. Then, the data fields at the fourth and fifth line from left to right include released date, released time, released technician, and released status, respectively. At last, there are two command buttons for this form from left to right: alert and exit. Actually, the LIS temporary stores the linked data in the SM table of LIS database. Consequently, the HSMS engine will poll the previous SM table every six minutes and transmit the specific message to the specified cell phone system one by one.

It is a challenge for laboratory worker to technically review all the remarkable laboratory results and decide to transmit an SM to the corresponding clinical doctors. Therefore, it is mandatory for the quality assurance committee to properly determine which outstanding laboratory results should be notified, the value of normal range of the laboratory results, the value of the alerting boundary of the laboratory results, etc.

Furthermore, some of the normal ranges of the laboratory results depend on the patient information, for example, sex, age, race, etc. It is manifest that if we are trying to add additional rules inside the information system to catch up with higher medical quality, the complexity of the technical implementation and average expense will increase exponentially.

The rate of abnormal laboratory report alerting cases was approximately 0.00398% from February 2006 to August 2008. That is, we found 2,060 abnormal cases among all laboratory specimen reports, from an average of 1,786,499 rows per month, during this specific execution period. Comparing with Bradshaw’s CLAS outcomes in 1989 [2], NTUH also promoted an average alert response time drop to around 3.6 hours when a review of alert conduct was integrated with laboratory result reviews, and to around 0.1 hour after installation of a short message service to notify hospital staff of the presence of new alerts.

Intuitively, most information engineers will adopt some of the latest mature information technologies to their implemented systems. After nearly 30 years of system utilization at the NTU hospital, the engineers of the Information Systems Office continuously identify some optimal solution to integrate most used systems. The main achievements for our proposed DiHOSE methodology and both of the HSMS and HMAS systems at least include seamless system integration with lower cost, quick system extension for implementing the SMS, promotion of health service quality for laboratory tests, emergent alerts for abnormal patient laboratory results, and easy maintenance for current and future system programmers.

## 6. Limitations and Future Work

Seeing that the crucial role limitation of the SMS system API is supported by the particular telecommunication companies, it is impossible for HMAS to process the acknowledgement message from the receiver. That is, the existing SM service is one-way from sender to receiver and does not support any acknowledgement mechanism for securing the successful transmission. If the telecommunication companies enthusiastically encourage two-way SMS system APIs, then the healthcare server quality will be improved as Kuperman, *et al.* [8] recommended in 1997. In order to noticeably enhance the healthcare quality, it is a necessary for NTUH to include more information technologies, for example, e-mail, to specially use numerous communication pipes to alert the physicians as described in [9–12]. Furthermore, the progressive and refined clinical decision support system like the functions mentioned in outstanding works [13–15] can be implemented after NTUH has well-established its DiHOSE.

On the other hand, it is difficult to find any other prevailing international standards related to both of the SMS and healthcare [16,20,21] and we can follow up or revise standards to adapt to healthcare enterprise usage. If an information system can successfully adopt appropriate standards to construct its framework, then the standardized message passing mechanism could be properly defined.

For example, the DiHOSE applies HL7 standard to specify the sufficient solicitation messages and integrate the HSMS engine without much work. Because it has been demonstrated very fruitful in [17–19], it is very convenient for disease surveillance to furnish the HSMS with DiHOSE and alert about special diseases in a timely manner within the healthcare enterprise, even expanding this country wide and integrated with the information systems at the Center for Disease Control (CDC).

## 7. Conclusions

Regardless how complex the information systems are, the clinical users inevitably submit new requirements to professionally assist the medical services based on their clinical services environment. Furthermore, some slight alterations of the functions or flows of an information system will constantly upgrade the clinical quality of medical services. It successfully develops the healthy dependence of the available service of information systems in the healthcare circumstances. The latest cell phone message alerting subsystem, a case study, in NTUH proved that the DiHOSE can integrate the user required functions without much work. We also inferred that both HSMS and DiHOSE can effectively generalize and definitely include formal request demands. Furthermore, the HMAS lets physicians be notified by an instant short message which includes some required and important laboratory information for a specific critical patient. Our solution would accomplish most of the healthcare monitoring requisitions and act as a flawless solution for intended healthcare notifying processing.
